# Reliability of a Robotic Knee Testing Tool to Assess Rotational Stability of the Knee Joint in Healthy Female and Male Volunteers

**DOI:** 10.1186/s40798-020-00266-7

**Published:** 2020-08-03

**Authors:** Samantha Beckley, Shaun Stinton, Maia Lesosky, Alison September, Malcolm Collins, Thomas Branch, Mike Posthumus

**Affiliations:** 1 Division of Exercise Science and Sports Medicine, Department of Human Biology, Faculty of Health Sciences, University of Cape Town, PO Box 115, Cape Town, Newlands 7700 South Africa; 2 End Range of Motion Improvement, Atlanta, GA USA; 3 Department of Public Health and Family Medicine, Faculty of Health Sciences, University of Cape Town, Cape Town, South Africa; 4 The Sport Science Institute of South Africa, Newlands, South Africa

**Keywords:** Humans, Knee joint, Reproducibility of results, Articular arthrometry, Six degrees of freedom movement

## Abstract

**Background:**

Several clinical tests exist to assess knee laxity. Although these assessments are the predominant tools of diagnosis, they are subjective and rely on the experience of the clinician. The robotic knee testing (RKT) device has been developed to quantitatively and objectively measure rotational knee laxity. The purpose of this study was primarily to determine the intra-tester reliability of rotational knee laxity and slack, the amount of rotation occurring between the two turning points of the load deformation curve, measured by the RKT device and investigate the differences between female and male measurements.

**Methods:**

Ninety-one healthy and moderately active volunteers took part in the study, of which twenty-five participated in the reliability study. Tibial rotation was performed using a servomotor to a torque of 6 N m, while measurements of motion in all 6° of freedom were collected. Reliability measurements were collected over 5 days at similar times of the day. Intra-class correlation coefficient (ICC) values and standard error of measurement (SEM) were determined across the load deformation curves. Linear mixed effects modelling was used to further assess the reliability of the measurement of external and internal tibial rotation using features of the curve (internal/external rotational laxity and slack). Measurements of internal/external rotational laxity and slack were compared between the sexes using the Student *t* test.

**Results:**

Pointwise axial rotation measurements of the tibia had good reliability [ICC (2,1) 0.83–0.89], while reliability of the secondary motions ranged between poor and good [ICC (2,1) 0.31–0.89]. All SEMs were less than 0.3°. Most of the variation of the curve features were accounted for by inter-subject differences (56.2–77.8%) and showed moderate to good reliability. Comparison of the right legs of the sexes revealed that females had significantly larger amounts of internal rotation laxity (females 6.1 ± 1.3° vs males 5.6 ± 0.9°, *p* = 0.037), external rotation laxity (females 6.0 ± 1.6° vs males 5.0 ± 1.2°, *p* = 0.002) and slack (females 19.2 ± 4.2° vs males 16.6 ± 2.9°, *p* = 0.003). Similar results were seen within the left legs.

**Conclusions:**

Overall, the RKT is a reliable and precise tool to assess the rotational laxity of the knee joint in healthy individuals. Finally, greater amounts of laxity and slack were also reported for females.

## Key Points

- In the future, the robotic knee testing device (RKT) could potentially be a useful tool for measuring tibial rotation while collecting data from all 6 degrees of freedom.
- External and internal tibial rotation (primary motion) was measured with good reliability using the RKT while secondary motion measurements showed poor to good reliability.
- On average, females were found to have greater amounts of rotational laxity compared to males.
- Less force is required for tibial rotation in females in comparison to males.

## Background

Knee laxity tests are commonly performed by clinicians to diagnose injuries as well as to assist with treatment choices [1, 2]. Increased anterior knee laxity and internal tibial rotation, as well as decreased tibial external rotation, have been associated with an increased risk of anterior cruciate ligament (ACL) injury [3, 4]. These laxity measurements, together with other risk factors, could therefore also be used to identify high risk athletes and to inform decision making processes to prevent and reduce the risk of knee injuries, particularly ACL injuries [5–7]. Similarly, these rotational measurements are also useful for the diagnosis of knee ligament injuries, as previous studies have shown that injury/transection of the ACL, specifically the posterolateral bundles, and the anterolateral ligament affects measurements of rotational laxity [8, 9]. An earlier study by Branch et al. also suggested that robotic measurements of the knee including obtained characteristics of the load deformation curve, such as end point stiffness, may provide beneficial information for more accurate knee injury diagnosis [10]. Therefore, the availability of tools to measure these movements is important.

**Fig. 1:**
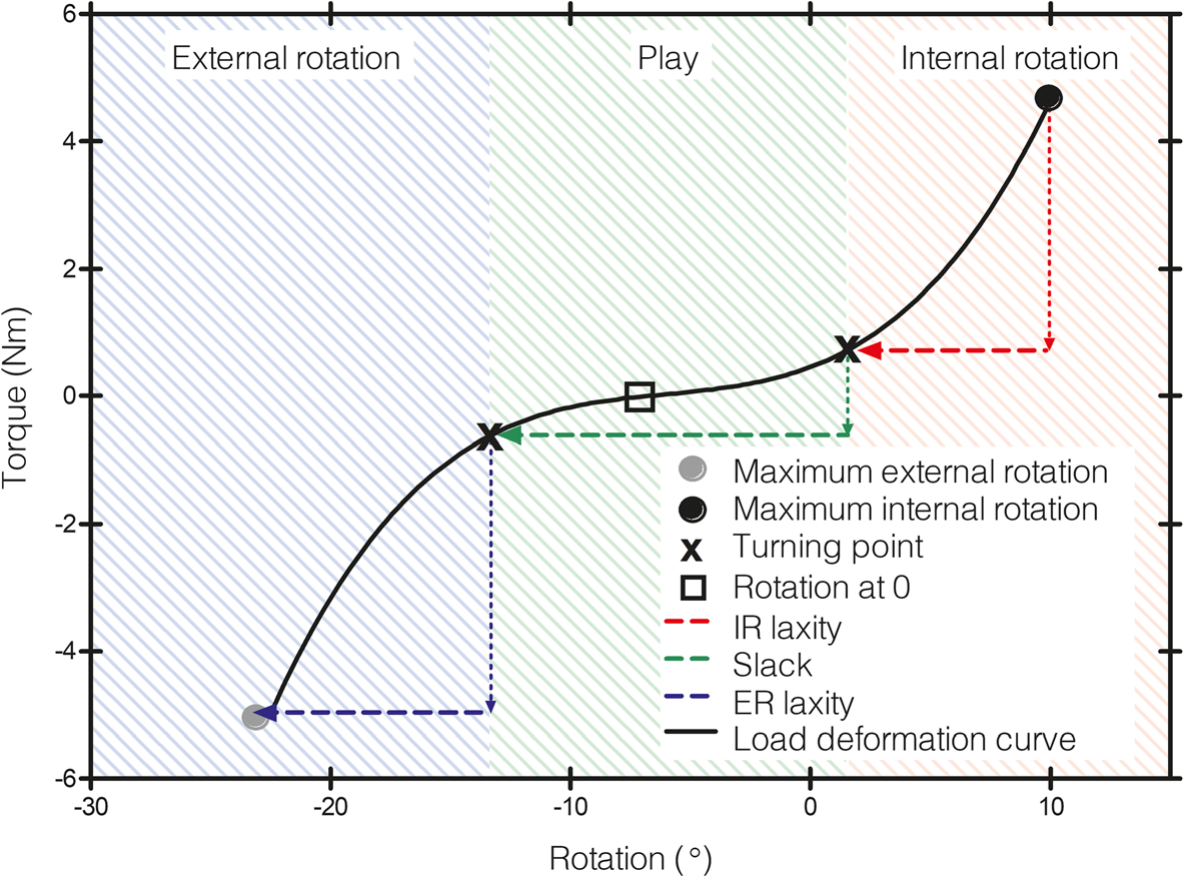
Features of the load deformation curve are depicted in the graph—maximum external rotation, maximum internal rotation, rotation at 0 and the calculated features internal rotation laxity, slack and external rotation laxity. The turning points are depicted which divide the curve into the three areas of external rotation, play and internal rotation

There are a growing number of devices developed to quantify knee laxity. The KT-1000® and KT-2000® are among the most popular and reliable arthrometers, which measure the anterior-posterior displacement of the tibia in relation to the femur [5, 11–13]. The technology to measure knee laxity has advanced technology such as electromagnetic sensors and triaxial accelerometers which have become more popular [14–16]. Additionally, an increasing amount of devices has been developed to measure rotational laxity [15–21]. The robotic knee testing (RKT) device used in this study was designed to measure internal and external tibial rotation in relation to the femur. This is achieved by applying a constant rotational force to the foot allowing the tibia to move freely. An electronic magnetic tracking device monitors movement, and data is collected from all 6 degrees of freedom, allowing the objective quantification of tibial rotation [10, 22]. As activities involving the knee occur in all 6 degrees of freedom, having this information will allow the clinician or researcher to gain a better understanding of how ligamentous laxity or ligament injury may affect an individual. Specifically, how certain ligaments may affect secondary motions [23]. Previous research has shown that measurements of coupled motions may allow a more accurate diagnosis of injuries [24]. To our knowledge, the reliability of a knee rotational measuring device in all 6 degrees of freedom has not been reported. Therefore, the primary purpose of this study was to determine the reliability of the RKT in measuring the internal and external rotation (primary motion) of the tibia in relation to the femur at 6 N m toque, as well as the reliability of movement within the other 5 degrees of freedom (secondary motions). In addition, the reliability, precision and sources of variation were assessed in the features of the load deformation curve. The specific mechanical properties analysed in this study were the internal and external rotational laxity (the amount of rotation occurring between the turning points at the maximal point of internal and external rotation, respectively), as well as slack (the amount of rotation occurring between the two turning points of the load deformation curve; Fig. 1). Interestingly, it has been suggested that females are at increased risk of sustaining an ACL injury compared to males [25–27]. The exact mechanisms underpinning the difference in the susceptibility still remain unknown. However, previous research has implicated differences in the structural and biomechanical properties of the knees [28]. In addition, it has further been described that females have a greater amount of anterior, varus, valgus and internal and external knee rotational laxity compared to males [3, 29, 30]. It has been hypothesized that this increased amount of knee laxity may put females at greater risk of injury [3, 5, 31]. Therefore, another aim of this study was to explore the differences in knee laxity measurements between females and males [32]. Based on findings from previous research, we hypothesise that females will have larger measurements of internal and external laxity as well as slack.

## Methods

Ninety-one moderately active participants between the ages of 20 and 48 were recruited from nearby fitness centres, social media or word of mouth. Participants were required to have no chronic or current (within the past 6 months) spinal cord injuries, as well as at least one healthy knee with no history of ACL injury. Prior to testing, each participant gave written informed consent in accordance with the Declaration of Helsinki. They also completed personal demographics, sporting history, injury history and medical history questionnaires. Ethical approval for the study was provided by the Research Ethics Committee of the Faculty of Health Sciences at the University of Cape Town, South Africa (reference number 859/2015).

Twenty-five of the above recruited participants with no history of knee ligament or meniscal injury took part in the reliability portion of this study. Rotational knee laxity measurements were completed daily, for 5 days over a maximum period of 7 days, at a similar time of the day.

**Fig. 2:**
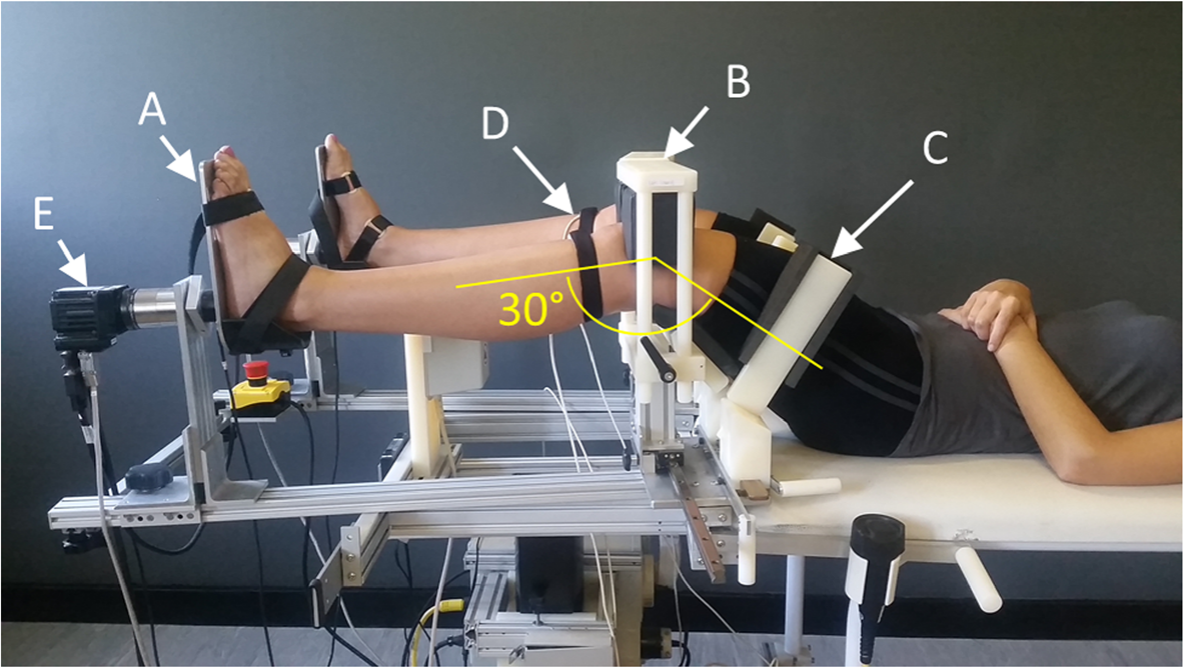
Participant setup in the robotic knee testing device with knees flexed at 30°, demonstrating the **a** foot plates, **b** knee clamps, **c** thigh clamps, **d** sensors on tibia and **e** servomotor

All setups and testing were carried out by SB, who had undergone limited setup and test execution training by the manufacturers. Participants were set up in the RKT device (Fig. 2) with the knee joint line over the thigh pads and the knee in approximately 30° of flexion. The feet were placed in the upright position and strapped tightly. The femur and patella were stabilised using the knee clamp which is tightened in place with 133 N of force. For further femur stabilisation, the thigh clamps were placed medially and laterally and tightened. In order to obtain a coordinate system for the stabilized femur, coordinates were taken at two fixed positions in the frame below the thigh pads as well as a third point in the centre of the knee clamp frame. Electromagnetic sensors were placed medially of the tibial tubercle on both legs. This allows for the comparison of the tibia coordinate system relative to that of the stabilized femur. The servomotor was responsible for the rotation on the foot plates, and these were rotated externally until a torque of − 6 N m was reached after which the foot plate changes direction. The foot plate continues to turn internally until a torque of 6 N m was reached. There are 3 cycles in total, including 4 external rotations and 3 internal rotations. Data was collected using the trakSTAR electromagnetic tracking system (trakSTAR™, Ascension Technology Corporation, Shelbourne, VT, USA) which has a static accuracy of 1.5 mm for sensor position and 0.5° for orientation. As a previous study showed the results from cycles are repeatable, only the third cycle was used for analysis [22].

This data was used to produce load deformation curves, which were further analysed to produce the features of the curve (Fig. 1). The maximum external rotation (ER) and internal rotation (IR) represent the largest external and internal rotation value achieved (in degrees). Rotation at 0 is the amount of rotation at 0 N m torque. The load deformation curve was divided into three sections: external rotation, internal rotation and area of play. Each of these areas is defined by a turning point in the curve. Turning points were determined by dividing the entire curve into two sections and fitting each with a cubic equation which we used to solve for the two points where the rate of change of torque becomes faster or slower than the rate of change in rotation. External rotation (ER) and internal rotation (IR) laxity is the amount of rotation that occurred between the respective external rotation or internal rotation turning points and the corresponding maximum rotation points. Slack is the amount of rotation which occurred in the play area, i.e. between the two turning points. For the purpose of this study, the calculated features of the curve—external laxity, internal laxity and slack—were focused on in order to examine the mechanical properties of knee joint.

Simple functional data analysis was completed to determine the pointwise mean, standard error of measurement (SEM) and intraclass correlation coefficient (ICC) along the entire load deformation curve in all 6 degrees of freedom for the rotational movement [33]. ICC (2,1) and SEM statistical analysis were performed using the R statistical software (version 3.4.2). ICC (2,1) values were determined using the *psych* package while the SEMs were calculated as described by Harvill (1991) [34], whereby variance was calculated using linear mixed effect modelling (LMM) which was based on the model used to examine the features of curve (described below) [35, 36]. The subject was included as a nested random effect, and knee (left or right side) was included as a fixed effect. ICC values were interpreted as follows: values under 0.5 were considered to show poor reliability, values between 0.5 and 0.75 demonstrated moderate reliability, values between 0.75 and 0.9 demonstrated good reliability and values above 0.9 showed excellent reliability [37].

LMM was used to determine the contribution of other factors to the variability of the features of the load deformation curve (internal rotational laxity, external rotational laxity and slack). All features were investigated using the same model, whereby three nested random effects were included in the models: knee (i.e. left or right knee) within subject and within day. Knee was additionally included as a fixed effect as exploratory analysis demonstrated trending significant differences between features on the right and left legs. Further variability was considered to be residual variability. The minimum detectable difference at the 90% confidence interval (MDC90) was calculated using the equation: MDC90 = 1.65 × SEM × √2 [38]. In order to determine the reliability of the features, the ICC (2,1) values were calculated in the R statistical software (version 3.4.2) using the *psych* package [35, 36].

Normal distribution of the curve features data was determined using Shapiro-Wilk normality tests. Two-tailed paired *t* tests or the related-samples Wilcoxon signed rank test was used to compare means of the curve features between the left and right legs. Data from uninjured legs of males and females were compared using independent *t* tests or Mann-Whitney *U* tests (data from the first day of testing was used for the reliability participants), depending on whether data was normally distributed. The left and right legs were analysed separately. Normality tests, *t* tests and Mann-Whitney *U* and the related-samples Wilcoxon signed rank tests were completed in the IBM SPSS Statistics (version 25) software. All graphs were produced using GraphPad Prism (version 5.03; GraphPad Software; San Diego, California).

## Results

The general characteristics of all recruited participants are shown in Table 1. The simple functional data analysis using ICC and SEM pointwise reliability of the axial rotation of the tibia and of the other 5 degrees of freedom was calculated from the load deformation curves (Supplementary Fig. S1). The ICC (2,1) axial rotation (Z rotation, primary motion; Supplementary Fig. S1A) values ranged between 0.83 and 0.89, while the SEM ranged between 0.14° and 0.18°. The ICC (2,1) abduction and adduction motion (Y rotation; Supplementary Fig. S1B) reliability scores ranged between 0.85 and 0.89, with SEMs between 0.14° and 0.17°. The ICC (2,1) flexion and extension motion (X rotation; Supplementary Fig. S1C) values ranged between 0.72 and 0.76, with SEMs between 0.21°and 0.23°. The ICC (2,1) for compression and distraction measurements (Z translation; Supplementary Fig. S1D), anterior and posterior movements (Y translation; Supplementary Fig. S1E) and medial and lateral translational measurements (X translation; Supplementary Fig. S1F) ranged between 0.42 and 0.57, 0.69 and 0.76, and 0.31 and 0.66, respectively. While the SEM ranges for Z, Y and X, translation measurements were 0.08 to 0.09°, 0.11 to 0.20° and 0.10° and 0.26°, respectively.

**Table 1:** The general characteristics of all participants and the comparison of values between males and females

	Total	Female	Male	*p* value
	*n* = 91	*n* = 38	*n* = 53	
Age (years)	27.0 (24.0; 33.0)	26.0 (23.0; 31.0)	27.0 (25.0; 33.0)	0.333^a^
Height (cm)	174.3 ± 10.0	165.2 ± 6.0	180.7 ± 6.7	0.000
Weight (kg)	75.0 (61.4; 82.4)	60.9 (57.9; 68.2)	80.6 (75.0; 89.6)	0.000^a^
BMI (kg.m2)	23.9 (22.2; 25.8)	22.8 (20.9; 24.7)	24.3 (23.2; 26.0)	0.006^a^
Dominant leg (% right)	84.6	84.2	84.9	0.576^b^

^a^*p* values calculated using Mann-Whitney *U* test

^b^Chi-squared test

Since the residual variance ranged from 2.2 to 3.2%, most of the variance of the three reported features of the load deformation curve could be explained by inter-subject (56.2 to 73.8%), left or right knee, (8.0 to 16.6%) and day-to-day testing (16.0 to 23.7%), where knee was nested within subject, and subject was nested within day-to-day testing (Table 2). The standard error of measurement (square root of the residual error) for the three features of the curve was low ranging between 0.0° and 0.6°, while the calculated ICC (2,1) reliability values for each of the features ranged between 0.61 and 0.76.

**Table 2:** Factors explaining variation, standard error of measurement (SEM), minimum detectable difference at the 90% confidence interval (MDC_90_), total variance and reliability of the features of the load deformation curve

Features	Variance components							Reliability score
	Subjects (% of variance)	Knee (% of variance)	Day (% of variance)	Residual (% of variance)	SEM (°)	MDC_90_	Total variance	ICC (2,1)
ER laxity	1.1 (70.5)	0.1 (8.4)	0.3 (17.9)	0.1 (3.2)	0.0	0.1	1.6	0.74
Slack	9.0 (73.8)	1.0 (8.0)	1.9 (16.0)	0.3 (2.2)	0.6	1.5	12.2	0.76
IR laxity	0.6 (56.2)	0.2 (16.6)	0.3 (23.7)	0.0 (3.2)	0.2	0.4	1.1	0.61

The general characteristics of the female and male participants are summarised in Table 1, and the comparison of the right and left leg measurements between females and males is shown in Table 3. On average, females showed greater amounts of slack (left 18.6 ± 3.5°; right 19.2 ± 4.2°) than males (left 16.6 ± 3.1°; right 16.6 ± 2.9°, *p* = 0.004 and 0.002, respectively, Table 3). Similarly, in females, the average amount of external laxity (left 5.9 ± 1.3°; right 6.0 ± 1.6°) was greater than males (left 5.1 ± 1.1°; right 5.0 ± 1.2°, *p* = 0.002, Table 3). A significant difference in amounts of internal laxity was only seen in the right leg of females (6.1 ± 1.3°) in comparison to those of the males (5.6 ± 0.9°, *p* = 0.037, Table 3). Although the females still had greater left leg measurements of internal laxity (5.9 ± 1.1°) in comparison to males (5.5 ± 1.3°, *p* = 0.181, Table 3), the difference was not found to be significant.

**Table 3:** Comparison of the features of the curve of males and females in the left and right legs. A total of 2 and 7 left leg and 3 and 12 right leg measurements did not meet the inclusion criteria for females and males, respectively, due to previous ligament or meniscus injuries

	Female	Male	*p* value	Power
Left leg	*n* = 36	*n* = 46		
External laxity (°)	5.9 ± 1.3	5.1 ± 1.1	0.002	0.80
Slack (°)	18.6 ± 3.5	16.6 ± 3.1	0.004^a^	0.70
Internal laxity (°)	5.9 ± 1.1	5.5 ± 1.3	0.181^a^	0.30
Right leg	*n* = 35	*n* = 41		
External laxity (°)	6.0 ± 1.6	5.0 ± 1.2	0.002	0.90
Slack (°)	19.2 ± 4.2	16.6 ± 2.9	0.003	0.90
Internal laxity (°)	6.1 ± 1.3	5.6 ± 0.9	0.037	0.40

It is interesting to note that, during analysis, a consistent difference was found between the right and left legs of individuals. Investigation into the setup of the device found a consistently greater rotation of ~ 3.5° of the right frame where the leg rests on the thigh pad in comparison to the left frame. Analysis demonstrated significant differences in the maximal external and internal rotation measurements between the right and left legs, but these differences were less apparent when comparing the calculated features of the curve (Supplementary Fig. 2).

## Discussion

It is important for testing equipment to provide reliable measurements to allow for confidence in reporting and interpreting these measurements [33]. The ICC, SEM and MDC values provided in this paper demonstrate the reliability of the RKT in testing rotational knee laxity. The pointwise measurement of the primary motion, axial rotation (Z-rotation) and the measurement of the curve feature, slack showed good reliability.

The ICC (2,1) value of the measurement of the primary motion of the test, tibial axial rotation (Z rotation), was determined to have good reliability according to the interpretation of ICC values by Portney and Watkins [37]. The remaining 5 degrees of freedom are considered to be the secondary motions and had varying reliability scores. The measurement of abduction and adduction (Y rotation) was shown to have good reliability with small SEMs, whereas measurements of flexion and extension (X rotation) had similar SEMs with moderate to good reliability, as the ICC (2,1) scores decreased during internal tibial rotation. On average, the translational measurements had lower ICC outcomes than the rotational measurements. The highest scoring measurement was the anterior and posterior movement (Y translation), which was considered to have moderate to good reliability. The compression and distraction (Z translation) and medial and lateral (X translation) measurements showed poor to moderate reliability over the period of external and internal rotation of the tibia but had small SEMs. These SEMs suggest that the measurements of the RKT are precise while the lower ICC scores are likely a result of the small amount of movement that occurs in these planes, together with the small amount of intersubject variability. As the calculation of ICC (2,1) values is determined by the ratio of the between between-subjects mean square (BMS), error mean square (EMS) and trial mean square (TMS), a small BMS will affect the ICC values and result in a decrease in the reliability score [39]. For this reason, it is important to take into account the SEM values together with the ICC score. The measurements of these secondary motions may be more useful in the diagnosis of injuries, whereby this additional informational may provide a more holistic picture of knee laxity following an injury and allowing for a more accurate diagnosis. For example, it is reasonable to hypothesise that an ACL injury may not only result in a greater amount of axial rotation but may also result in increased measures of anterior translation, flexion and abduction. Future studies obtaining measurements in all 6 degrees of freedom from participants with soft tissue knee injuries using the RKT could potentially provide more information as to how the RKT could be used as part of a diagnostic tool by clinicians in the future.

In previous research using a similar knee rotation device, Branch et al. found the device to produce excellent reliability scores for the measurement of axial rotation of the tibia. The slightly lower scores found during this study may be explained by the different technology used in the earlier version of the RKT device. The version used for the current study used electromagnetic sensors, whereas the earlier version used by Branch et al. measured rotation using the encoder count in the servomotor [22].

Previous studies have shown that the levels of sensitivity, specificity and accuracy increased as the amount of torque or force increased [40]. This is in agreement with the results of our simple functional data analysis showing the pointwise measurement of axial rotation at the larger torque values was more reliable than closer to 0 N m. Our ICC values at the end points of axial rotation (Z rotation) are lower than previously reported values [15, 16, 20, 21]. A possible reason for this may be the limited training the tester received or the experimental setup. The reliability testing of these studies took place over a single testing session where multiple measurements were taken or over 2 days, whereas the reliability data for current study was collected over 5 days. To our knowledge, there is no other published reliability scores for the secondary motion of the knee during rotational laxity testing and therefore, these scores cannot be compared to previous work.

The majority of the variation in this study was due to the subject to subject differences for all features of the curve. Furthermore, the reliability for the features based on the ICC (2,1) scores were interpreted to be moderate to good [37]. Therefore, as the correct source of variance, subject differences, was being measured, as opposed to side-to-side differences or day-to-day variations, the tester is reliably able to measure the true difference (i.e. intersubject variation). It should be noted that between 16% and 23.7% of the variance was accounted for by daily variation. There is a lack of literature available to use for comparison with these values, to determine whether this is similar to the variation found in other studies. Although this may be a typical amount of day-to-day variation, these systematic errors could also be explained by the limited amount of training the tester underwent prior to the current study. Additionally, the low percentages of residual variance suggest most of the variance was accounted for within the three previously mentioned factors. Internal rotation laxity measurement had the lowest percentage of variation due to subject; therefore, these values should be interpreted with some caution. External and internal rotational laxity had moderate reliability scores while slack was considered to have good reliability. Similarly to the ICC (2,1) scores for the translational movement, internal and external laxity had lower BMS values likely resulting in a lower ICC score. The low standard error together with the low SEMs and the small MDC values for the measurement of axial rotation suggest that the determined means for the features of the curve provide a precise and reliable data for a healthy, uninjured population. Therefore, these measurements (internal and external rotational laxity as well slack) may be useful for diagnostics or measurement of a patient’s laxity over time such as pre- and post-surgery or pre- and post- rehabilitation. It may be of interest in future research to repeat the testing using other similar knee rotation devices which have previously been validated and compare the LMM results of these other devices to those of the RKT [16, 21]. Such results would allow the comparison of the precision of the devices and would assist in understanding whether the variation in readings were a result of subject to subject differences.

The results also showed that females had significantly greater average or median measurements of internal and external laxity. These results coincide with numerous earlier studies confirming females have greater amounts of tibial external rotation than males [3, 29, 30]. An additional finding in this study was that females had a greater amount of slack than males. This suggests that less force is required to create a greater amount of tibial rotation compared to males. As females are also more susceptible to ACL injuries and greater measurements of internal laxity have been associated with risk of ACL rupture, this difference in laxity profile between females and males may partially explain why females are at greater risk. Future studies investigating the difference of slack measurements between participants with a history of ACL or other ligaments injuries and those of healthy can help to determine whether this measurement may be useful tool to include in an injury risk profile.

A limitation of this study was the noted difference in the setup of the right leg in comparison to the left leg. This variance may explain the consistent difference in the measurements of the right and left leg, although this did not seem to affect the reliability as the ICC scores between the left and right legs were similar. Furthermore, these differences were less apparent when comparing the calculated measurements (i.e. internal and external laxity as well as slack). Additionally, no electromagnetic sensors were placed on the thighs to account for any movement of the femur. Although unpublished data suggests that only small amounts of anterior-posterior (2 mm) and medial-lateral (3.5 mm) translation occurs, future studies would benefit from including the movement of the femur into the analysis of knee movement. As a systemic difference was found between day-to-day testing, an additional improvement to the subject setup in the RKT device may include a more automated setup allowing for less error when retesting patients or subjects.

## Conclusion

The RKT can be used to measure rotation of the knee while recording measurements in all 6 degrees of freedom. The primary motion, external and internal rotation, was found to have good reliability. Additionally, the features of the curve can be used for the assessment of knee laxity in future studies. Although reliability ranged from moderate to good, the small SEM and MDC values, as well as the measurement of the true source of variation, subject to subject differences demonstrate the usefulness of these features. The reliability of the measurement of secondary motions varied between poor to good. The translational motions with poor reliability should be interpreted with caution. Using the features of the curve to compare tibial rotation between females and males showed greater amounts of laxity and a greater amount of slack in females. Future work should include the improvement of the measurement of translational movements during testing and the reliability scores of the curve features.

## Supplementary information

**Additional file 1: Figure S1.** ICC(2,1) (dotted) plotted on the right y-axis and mean rotation (solid) and translation with standard error of measurement (shaded) plotted on the left y-axis. Standard error of measurement ranges are shown in the top left corner. Data is shown pointwise for RKT measurements of tibia movement in 6 degrees of freedom during external and internal rotation of the tibia. A) External and internal rotation, B) abduction and adduction, C) flexion and extension, D) compression and distraction, E) anterior and posterior translation F) medial and lateral translation. **Figure S2.** Scatter plots for the features of the curve comparing the left (circles) and right (crosses) legs within the features of the curve, A) external rotation laxity, B) internal rotation laxity and C) slack. Means of the left and right legs were statistically analysed using a paired t test or the Related-Samples Wilcoxon Signed Rank test (*=p<0.05). **Table S1.** Individual data of load deformation curve features - Each row contains the mean, standard deviation and the range of load deformation curve features (in columns) for both legs for each participant.

## Data Availability

The datasets used and/or analysed during the current study are available from the corresponding author on reasonable request.

## Abbreviations

ACL: Anterior cruciate ligament
BMS: Between-subjects mean square
ER: External rotation
EMS: Error mean square
ICC: Intraclass correlation coefficient
IR: Internal rotation
LMM: Linear mixed modelling
MDC_90_: Minimum detectable difference at the 90% confidence interval
RKT: Robotic knee testing
SEM: Standard error of measurement
TMS: Trial mean square
