# Supplementary material for: Reliability of a Robotic Knee Testing Tool to Assess Rotational Stability of the Knee Joint in Healthy Female and Male Volunteers
Source: Sports Med Open. 2020 Aug 3;6:33. doi: 10.1186/s40798-020-00266-7 (PMC7399727; doi:10.1186/s40798-020-00266-7)
Supplement: Supplementary file 1 — Additional file 1: Figure S1. ICC(2,1) (dotted) plotted on the right y-axis and mean rotation (solid) and translation with standard error of measurement (shaded) plotted on the left y-axis. Standard error of measurement ranges are shown in the top left corner. Data is shown pointwise for RKT measurements of tibia movement in 6 degrees of freedom during external and internal rotation of the tibia. A) External and internal rotation, B) abduction and adduction, C) flexion and extension, D) compression and distraction, E) anterior and posterior translation F) medial and lateral translation. Figure S2. Scatter plots for the features of the curve comparing the left (circles) and right (crosses) legs within the features of the curve, A) external rotation laxity, B) internal rotation laxity and C) slack. Means of the left and right legs were statistically analysed using a paired t test or the Related-Samples Wilcoxon Signed Rank test (*=p<0.05). Table S1. Individual data of load deformation curve features - Each row contains the mean, standard deviation and the range of load deformation curve features (in columns) for both legs for each participant. [file 40798_2020_266_MOESM1_ESM.docx]

# Supplementary figures


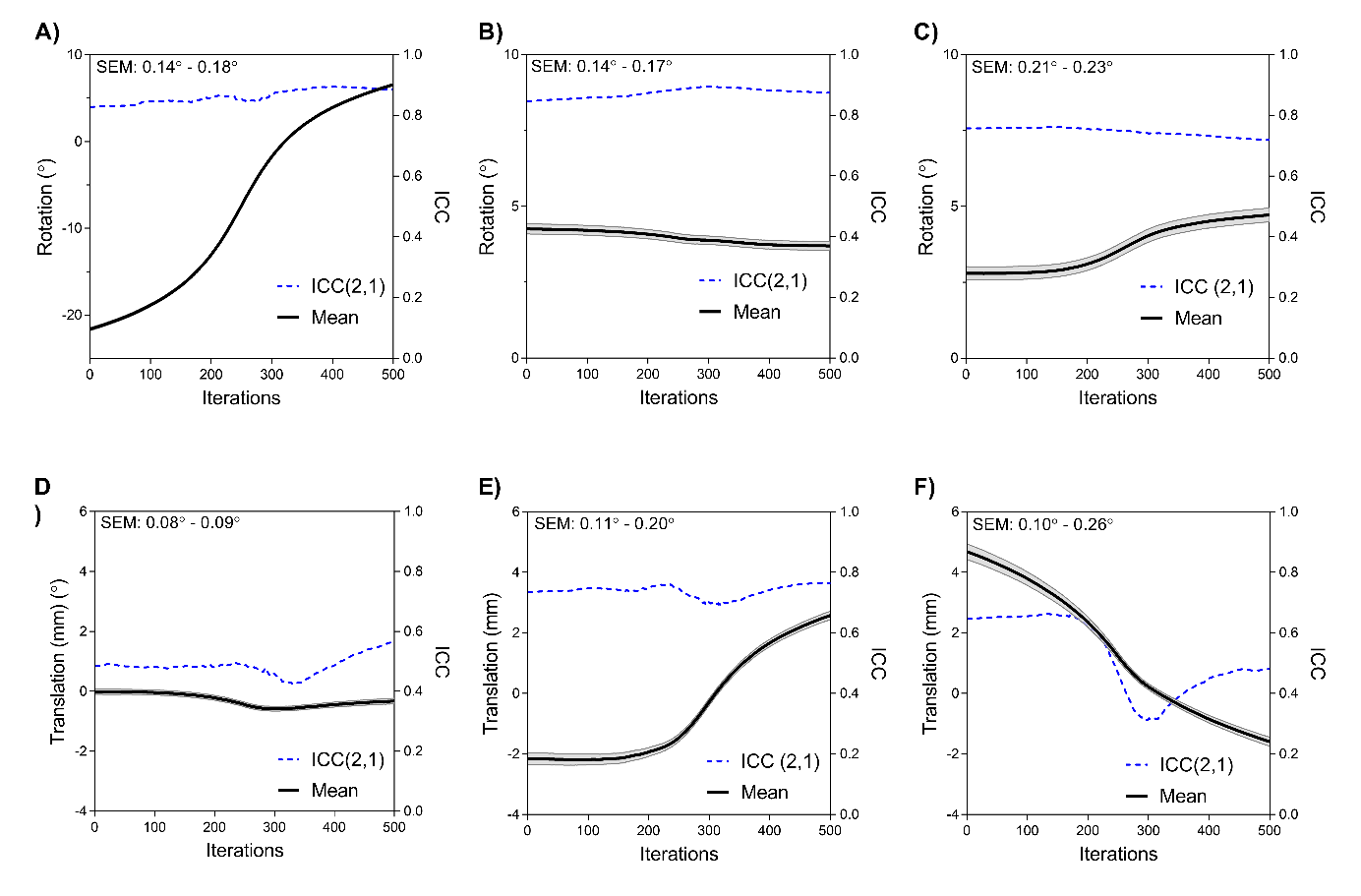


**Figure S1:** ICC(2,1) (dotted) plotted on the right y-axis and mean rotation (solid) and translation with standard error of measurement (shaded) plotted on the left y-axis. Standard error of measurement ranges are shown in the top left corner. Data is shown pointwise for RKT measurements of tibia movement in 6 degrees of freedom during external and internal rotation of the tibia. A) External and internal rotation, B) abduction and adduction, C) flexion and extension, D) compression and distraction, E) anterior and posterior translation F) medial and lateral translation.





**Figure S2:** Scatter plots for the features of the curve comparing the left (circles) and right (crosses) legs within the features of the curve, A) external rotation laxity, B) internal rotation laxity and C) slack. Means of the left and right legs were statistically analysed using a paired t test or the Related-Samples Wilcoxon Signed Rank test (*=p<0.05).

# Supplementary table data

**Table S1:** Individual data of load deformation curve features - Each row contains the mean, standard deviation and the range of load deformation curve features (in columns) for both legs for each participant.

|  | Max. External Rotation | | Max. Internal Rotation | | Rotation at 0 N m | | External Rotation laxity | | Internal rotation laxity | | Slack | |
| --- | --- | --- | --- | --- | --- | --- | --- | --- | --- | --- | --- | --- |
|  | L | R | L | R | L | R | L | R | L | R | L | R |
| 1 | -20.9 ± 2.2 | -30.0 ± 1.4 | 7.0 ± 1.7 | 1.2 ± 0.8 | -8.4 ± 1.3 | -14.3 ± 1.4 | 5.5 ± 0.7 | 6.1 ± 0.3 | 5.2 ± 0.5 | 6.1 ± 0.3 | 17.1 ± 1.9 | 18.9 ± 0.9 |
|  | (-24.0 - -16.8) | (-32.8 - -28.7) | (4.7 - 9.5) | (0.0 - 2.9) | (-10.3 - -6.0) | (-16.6 - -12.4) | (4.3 - 6.3) | (5.8 - 6.8) | (4.1 - 5.6) | (5.7 - 6.5) | (13.5 - 18.7) | (17.7 - 20.9) |
| 2 | -17.4 ± 2.1 | -23.6 ± 1.0 | 13.6 ± 1.2 | 10.5 ± 0.9 | -2.6 ± 0.5 | -6.5 ± 1.7 | 6.2 ± 0.5 | 6.9 ± 0.3 | 5.6 ± 0.4 | 6.1 ± 0.3 | 19.1 ± 1.0 | 21.0 ± 0.7 |
|  | (-20.5 - -14.2) | (-25.5 - -22.0) | (11.7 - 15.4) | (9.2 - 11.8) | (-3.4 - -1.7) | (-8.8 - -3.7) | (5.3 - 6.7) | (6.2 - 7.4) | (4.8 - 6.2) | (5.8 - 6.6) | (17.2 - 20.3) | (19.7 - 22.0) |
| 3 | -26.7 ± 1.1 | -32.6 ± 1.3 | 9.7 ± 0.7 | 11.2 ± 0.4 | -9.1 ± 1.2 | -9.3 ± 1.2 | 7.5 ± 0.4 | 8.8 ± 0.4 | 6.4 ± 0.3 | 7.9 ± 0.4 | 22.3 ± 1.0 | 26.8 ± 1.1 |
|  | (-28.4 - -25.2) | (-34.9 - -29.5) | (8.7 - 11.1) | (10.5 - 12.3) | (-11.4 - -7.5) | (-10.8 - -6.9) | (6.9 - 8.1) | (7.9 - 9.3) | (5.9 - 7.0) | (7.3 - 8.7) | (20.8 - 24.0) | (24.0 - 28.2) |
| 4 | -22.5 ± 2.2 | -27.5 ± 1.5 | 12.8 ± 1.3 | 8.7 ± 1.2 | -3.6 ± 0.6 | -7.9 ± 0.5 | 6.8 ± 0.7 | 6.9 ± 0.5 | 6.9 ± 0.6 | 7.3 ± 0.4 | 21.6 ± 2.0 | 22.0 ± 1.6 |
|  | (-25.3 - -19.4) | (-29.2 - -24.6) | (10.8 - 14.3) | (7.2 - 10.4) | (-4.6 - -2.7) | (-8.6 - -7.1) | (5.9 - 7.7) | (6.1 - 7.5) | (6.0 - 7.7) | (6.5 - 7.7) | (18.9 - 24.2) | (19.5 - 24.2) |
| 5 | -27.6 ± 1.6 | -32.8 ± 1.1 | 7.7 ± 0.9 | 4.3 ± 0.8 | -8.8 ± 1.0 | -12.9 ± 0.7 | 6.5 ± 0.3 | 6.8 ± 0.4 | 7.1 ± 0.2 | 7.4 ± 0.3 | 21.6 ± 0.9 | 22.6 ± 0.8 |
|  | (-29.9 - -24.8) | (-35.0 - -31.5) | (6.5 - 9.3) | (3.0 - 5.3) | (-10.3 - -7.1) | (-14.1 - -11.8) | (5.9 - 7.1) | (5.8 - 7.4) | (6.8 - 7.4) | (6.8 - 8.2) | (20.2 - 23.0) | (20.9 - 23.8) |
| 6 | -24.3 ± 1.1 | -25.2 ± 0.6 | 12.2 ± 1.0 | 9.8 ± 1.0 | -4.1 ± 0.7 | -7.1 ± 0.9 | 7.4 ± 0.2 | 6.9 ± 0.2 | 7.0 ± 0.3 | 6.6 ± 0.1 | 22.0 ± 0.5 | 21.5 ± 0.5 |
|  | (-25.9 - -22.8) | (-26.1 - -24.5) | (11.0 - 13.7) | (8.8 - 11.4) | (-5.5 - -3.1) | (-8.1 - -6.0) | (7.0 - 7.7) | (6.5 - 7.1) | (6.6 - 7.5) | (6.2 - 6.8) | (21.0 - 22.5) | (20.7 - 22.3) |
| 7 | -19.9 ± 1.4 | -25.5 ± 0.7 | 12.3 ± 1.4 | 4.1 ± 1.1 | -2.8 ± 1.1 | -8.4 ± 0.9 | 6.8 ± 0.3 | 6.2 ± 0.3 | 6.2 ± 0.4 | 5.7 ± 0.4 | 19.1 ± 0.7 | 17.7 ± 0.7 |
|  | (-21.8 - -17.8) | (-26.5 - -24.3) | (10.3 - 14.9) | (2.1 - 5.4) | (-4.4 - -0.7) | (-10.0 - -7.1) | (6.3 - 7.5) | (5.7 - 6.5) | (5.6 - 6.9) | (5.1 - 6.4) | (17.6 - 20.0) | (16.6 - 18.8) |
| 8 | -20.3 ± 2.2 | -27.0 ± 0.8 | 6.2 ± 1.0 | 0.6 ± 0.7 | -7.1 ± 1.4 | -12.1 ± 1.0 | 5.5 ± 0.3 | 5.5 ± 0.2 | 5.0 ± 0.2 | 5.4 ± 0.4 | 15.9 ± 0.8 | 16.6 ± 0.5 |
|  | (-23.8 - -17.6) | (-28.4 - -25.9) | (4.8 - 7.7) | (-0.6 - 1.8) | (-9.5 - -5.0) | (-13.2 - -10.0) | (5.0 - 6.0) | (5.1 - 5.7) | (4.8 - 5.3) | (4.9 - 6.0) | (14.8 - 17.6) | (15.9 - 17.6) |
| 9 | -20.0 ± 1.6 | -26.9 ± 1.9 | 9.8 ± 1.7 | 2.1 ± 0.5 | -5.0 ± 1.1 | -10.4 ± 1.4 | 6.2 ± 0.6 | 5.3 ± 0.9 | 5.6 ± 0.6 | 5.6 ± 0.6 | 18.0 ± 1.7 | 17.6 ± 1.5 |
|  | (-22.7 - -18.3) | (-28.4 - -20.7) | (6.2 - 11.4) | (1.2 - 3.1) | (-7.1 - -3.6) | (-12.5 - -8.1) | (5.2 - 6.9) | (3.0 - 6.0) | (4.5 - 6.9) | (3.9 - 6.1) | (14.8 - 19.7) | (13.8 - 20.7) |
| 10 | -26.2 ± 6.4 | -32.7 ± 1.0 | 12.7 ± 2.1 | 7.6 ± 0.9 | -4.2 ± 1.9 | -7.9 ± 1.2 | 7.3 ± 1.4 | 7.4 ± 0.5 | 7.5 ± 1.6 | 8.4 ± 0.5 | 23.5 ± 4.1 | 24.1 ± 0.8 |
|  | (-42.8 - -20.1) | (-34.2 - -31.0) | (9.7 - 17.7) | (5.7 - 9.1) | (-7.5 - -2.2) | (-11.6 - -6.6) | (5.7 - 10.1) | (6.6 - 8.3) | (5.5 - 10.9) | (7.4 - 9.0) | (18.8 - 35.7) | (22.5 - 25.5) |
| 11 | -18.1 ± 1.5 | -25.1 ± 2.1 | 10.7 ± 0.9 | 6.9 ± 1.6 | -2.7 ± 0.5 | -8.1 ± 1.9 | 4.9 ± 0.3 | 5.5 ± 0.2 | 6.1 ± 0.4 | 6.7 ± 0.8 | 17.7 ± 0.7 | 19.7 ± 1.1 |
|  | (-20.3 - -16.4) | (-29.0 - -23.0) | (9.2 - 11.8) | (5.1 - 9.6) | (-3.7 - -2.3) | (-10.5 - -5.0) | (4.5 - 5.6) | (5.1 - 5.7) | (5.5 - 6.6) | (5.4 - 7.7) | (16.4 - 18.9) | (18.1 - 21.8) |
| 12 | -19.5 ± 3.5 | -26.3 ± 1.4 | 8.6 ± 1.6 | 7.3 ± 1.0 | -4.6 ± 1.1 | -7.9 ± 1.4 | 5.6 ± 1.1 | 6.8 ± 0.3 | 5.6 ± 0.9 | 6.4 ± 0.4 | 16.8 ± 2.8 | 20.1 ± 0.6 |
|  | (-23.8 - -13.1) | (-28.3 - -24.5) | (5.8 - 10.6) | (5.6 - 8.9) | (-7.7 - -3.4) | (-10.7 - -5.3) | (3.6 - 6.7) | (6.3 - 7.4) | (3.9 - 6.6) | (5.6 - 7.1) | (11.6 - 19.9) | (19.3 - 21.4) |
| 13 | -22.0 ± 1.9 | -25.3 ± 0.5 | 7.7 ± 0.7 | 2.7 ± 0.8 | -5.6 ± 1.1 | -9.2 ± 0.8 | 5.7 ± 0.3 | 5.3 ± 0.2 | 6.1 ± 0.4 | 5.9 ± 0.3 | 17.7 ± 0.9 | 16.8 ± 0.4 |
|  | (-24.3 - -18.5) | (-26.6 - -24.8) | (6.3 - 8.6) | (1.1 - 3.6) | (-7.7 - -3.8) | (-10.6 - -8.1) | (5.0 - 6.2) | (5.0 - 5.8) | (5.5 - 6.9) | (5.3 - 6.1) | (16.0 - 18.9) | (16.3 - 17.6) |
| 14 | -16.8 ± 1.4 | -20.4 ± 1.2 | 9.1 ± 0.4 | 4.0 ± 0.8 | -1.9 ± 0.8 | -6.2 ± 0.5 | 5.4 ± 0.3 | 4.9 ± 0.4 | 4.9 ± 0.3 | 4.8 ± 0.4 | 15.5 ± 0.8 | 14.7 ± 1.1 |
|  | (-19.4 - -14.9) | (-21.5 - -17.7) | (8.4 - 9.8) | (2.8 - 5.3) | (-3.4 - -1.0) | (-6.8 - -5.0) | (4.9 - 5.9) | (4.1 - 5.3) | (4.4 - 5.4) | (3.8 - 5.1) | (14.5 - 16.9) | (12.6 - 15.9) |
| 15 | -21.6 ± 2.1 | -28.1 ± 1.4 | 8.9 ± 0.9 | 5.1 ± 0.4 | -6.4 ± 0.7 | -10.1 ± 0.9 | 6.3 ± 0.7 | 6.9 ± 0.3 | 5.5 ± 0.6 | 6.4 ± 0.2 | 18.5 ± 1.6 | 19.7 ± 0.5 |
|  | (-25.1 - -18.2) | (-30.4 - -26.5) | (7.6 - 10.3) | (4.5 - 6.0) | (-7.4 - -5.3) | (-11.5 - -8.3) | (5.5 - 7.5) | (6.4 - 7.5) | (4.6 - 6.8) | (6.1 - 6.8) | (16.4 - 21.4) | (19.0 - 20.9) |
| 16 | -15.5 ± 1.1 | -25.8 ± 1.6 | 12.6 ± 1.5 | 9.2 ± 0.9 | -2.2 ± 0.2 | -8.1 ± 0.9 | 6.0 ± 0.6 | 6.5 ± 1.0 | 4.7 ± 0.4 | 7.1 ± 0.5 | 17.3 ± 1.5 | 21.2 ± 0.8 |
|  | (-16.7 - -13.9) | (-27.5 - -22.9) | (9.6 - 14.2) | (7.8 - 10.6) | (-2.6 - -1.9) | (-9.6 - -6.2) | (5.1 - 6.7) | (4.8 - 7.8) | (3.9 - 5.1) | (6.3 - 7.9) | (14.4 - 18.7) | (19.8 - 22.4) |
| 17 | -19.0 ± 1.6 | -24.5 ± 1.9 | 8.8 ± 2.2 | 5.5 ± 0.9 | -2.4 ± 1.4 | -7.6 ± 0.9 | 4.4 ± 0.9 | 5.1 ± 0.4 | 5.6 ± 0.3 | 6.2 ± 0.5 | 17.2 ± 1.9 | 18.5 ± 1.2 |
|  | (-21.4 - -16.0) | (-27.3 - -22.4) | (6.2 - 12.7) | (4.3 - 6.6) | (-4.2 - 0.2) | (-9.0 - -5.4) | (3.0 - 6.0) | (4.5 - 5.9) | (5.2 - 6.0) | (5.2 - 7.0) | (14.7 - 20.5) | (16.6 - 20.4) |
| 18 | -16.7 ± 1.1 | -18.0 ± 1.0 | 6.4 ± 1.1 | 2.7 ± 0.9 | -2.6 ± 0.9 | -5.4 ± 0.7 | 4.4 ± 0.2 | 3.2 ± 0.4 | 4.8 ± 0.3 | 5.1 ± 0.4 | 13.9 ± 0.7 | 12.1 ± 1.3 |
|  | (-18.4 - -14.1) | (-19.0 - -15.3) | (4.4 - 8.3) | (1.6 - 4.1) | (-4.3 - -1.4) | (-6.2 - -3.7) | (4.1 - 4.7) | (2.4 - 3.8) | (4.3 - 5.4) | (4.2 - 5.7) | (12.6 - 14.9) | (10.6 - 14.5) |
| 19 | -15.8 ± 1.0 | -18.9 ± 4.6 | 9.8 ± 1.6 | 5.4 ± 1.6 | -1.0 ± 0.9 | -4.9 ± 2.0 | 4.8 ± 0.5 | 4.6 ± 1.1 | 5.3 ± 0.5 | 5.0 ± 1.1 | 15.4 ± 1.3 | 14.7 ± 3.0 |
|  | (-16.9 - -14.6) | (-24.5 - -14.1) | (6.8 - 11.4) | (3.1 - 8.2) | (-2.3 - 0.3) | (-8.8 - -3.3) | (3.9 - 5.4) | (3.3 - 6.5) | (4.5 - 6.1) | (3.7 - 6.8) | (13.0 - 16.8) | (11.0 - 19.1) |
| 20 | -18.1 ± 2.0 | -24.2 ± 1.9 | 6.3 ± 0.5 | 3.8 ± 0.6 | -4.1 ± 0.8 | -8.9 ± 0.8 | 4.6 ± 0.4 | 5.4 ± 0.5 | 4.9 ± 0.5 | 5.5 ± 0.4 | 14.8 ± 1.3 | 17.0 ± 1.4 |
|  | (-20.3 - -15.4) | (-25.6 - -20.5) | (5.4 - 7.0) | (2.9 - 4.6) | (-5.0 - -3.0) | (-10.3 - -7.9) | (4.1 - 5.1) | (4.5 - 6.1) | (4.4 - 5.6) | (4.7 - 5.9) | (13.3 - 16.5) | (14.3 - 18.3) |
| 21 | -19.4 ± 1.4 | -22.0 ± 2.0 | 6.6 ± 0.4 | 1.8 ± 1.0 | -4.6 ± 0.6 | -9.0 ± 0.8 | 4.9 ± 0.3 | 4.4 ± 0.5 | 5.5 ± 0.3 | 5.0 ± 0.5 | 15.5 ± 0.9 | 14.3 ± 1.4 |
|  | (-22.0 - -17.7) | (-25.8 - -19.6) | (5.8 - 7.3) | (0.3 - 3.3) | (-5.4 - -3.4) | (-10.5 - -8.3) | (4.5 - 5.5) | (3.7 - 5.0) | (5.2 - 6.2) | (4.0 - 5.8) | (14.5 - 17.2) | (12.2 - 16.0) |
| 22 | -24.7 ± 1.4 | -27.1 ± 1.2 | 2.9 ± 1.5 | 0.5 ± 2.0 | -11.0 ± 1.1 | -14.2 ± 1.0 | 5.3 ± 0.2 | 5.3 ± 0.6 | 5.4 ± 0.3 | 5.4 ± 0.4 | 16.8 ± 0.6 | 16.7 ± 1.3 |
|  | (-26.4 - -22.3) | (-28.9 - -25.6) | (1.2 - 5.0) | (-2.3 - 3.6) | (-12.9 - -9.0) | (-15.6 - -12.4) | (4.9 - 5.6) | (4.4 - 6.2) | (4.9 - 5.8) | (4.9 - 5.9) | (15.9 - 18.1) | (14.8 - 18.5) |
| 23 | -17.1 ± 1.5 | -21.0 ± 1.2 | 4.0 ± 0.9 | 1.0 ± 0.3 | -4.9 ± 1.0 | -8.7 ± 0.6 | 3.9 ± 0.2 | 3.9 ± 0.3 | 4.8 ± 0.3 | 4.9 ± 0.3 | 12.5 ± 0.4 | 13.2 ± 0.8 |
|  | (-19.2 - -14.6) | (-23.2 - -19.7) | (3.1 - 5.8) | (0.6 - 1.4) | (-6.6 - -3.7) | (-9.7 - -8.1) | (3.6 - 4.2) | (3.6 - 4.5) | (4.2 - 5.2) | (4.5 - 5.3) | (11.7 - 13.1) | (12.3 - 14.6) |
| 24 | -16.6 ± 1.1 | -22.1 ± 1.1 | 12.8 ± 1.6 | 8.6 ± 2.1 | -0.1 ± 1.2 | -4.9 ± 0.7 | 5.6 ± 0.6 | 5.9 ± 0.8 | 5.7 ± 0.5 | 5.9 ± 0.4 | 18.0 ± 1.5 | 18.7 ± 1.7 |
|  | (-18.0 - -14.6) | (-23.8 - -20.3) | (10.3 - 15.4) | (4.2 - 11.0) | (-3.7 - 1.5) | (-6.1 - -4.0) | (4.1 - 6.6) | (4.0 - 6.9) | (4.6 - 6.3) | (5.3 - 6.6) | (15.5 - 20.2) | (14.7 - 20.9) |
| 25 | -15.6 ± 1.6 | -17.1 ± 2.1 | 8.2 ± 1.3 | 5.1 ± 1.3 | -1.4 ± 1.2 | -5.6 ± 1.1 | 3.9 ± 0.5 | 4.3 ± 0.4 | 4.7 ± 0.6 | 4.2 ± 0.5 | 14.9 ± 1.6 | 13.7 ± 1.5 |
|  | (-17.9 - -13.1) | (-18.8 - -13.0) | (6.8 - 10.8) | (2.8 - 6.8) | (-2.7 - 0.7) | (-7.3 - -3.6) | (3.1 - 4.7) | (3.5 - 4.8) | (4.0 - 5.5) | (3.5 - 4.9) | (12.3 - 17.2) | (11.6 - 15.6) |
